# Quality changes in cocoyam flours during storage

**DOI:** 10.1002/fsn3.347

**Published:** 2016-02-17

**Authors:** Adewale Obadina, Hannah Ashimolowo, Ifeoluwa Olotu

**Affiliations:** ^1^ Departments of Food Science & TechnologyFederal University of AgricultureAbeokutaP.M.B. 2240Nigeria; ^2^ Department of Biotechnology and Food TechnologyUniversity of JohannesburgDoornfontein CampusP.O. Box 17011GautengSouth Africa

**Keywords:** Cocoyam, fermentation, parboiling, pasting properties, storage

## Abstract

Cocoyam tubers were subjected to two processing methods (fermentation and parboiling) and milled into flour. The samples were stored at 25°C, 35°C, and 45°C and analyzed at 2 weeks interval for 8 weeks for their nutritional, functional, and pasting properties. Data obtained were subjected to multiple analysis of variance. Storage period and its interactive effect with the processing methods had significant (*P* < 0.05) effects on all the nutritional composition of the flour except for the fat content. Water absorption index, wettability, and bulk density ranged from 1.94–2.72%, 25.30–135.50 sec, and 0.48–0.71 g/cm^3^, respectively. The entire pasting properties were significantly affected by the storage period while the interaction between storage temperature, processing methods, and storage period affected all the pasting properties except the peak time, peak, and breakdown viscosity. This study showed that processing methods storage periods and storage temperatures produced cocoyam flour with different quality characteristics.

## Introduction

Cocoyam (*Colocasia esculenta*) is an herbaceous perennial plant which is classified under the family Araceae and is mainly grown for their edible roots. A significant part of the world cocoyam production (three quarter) is from Africa with Nigeria and Ghana being the leading producers. After yam and cassava being roots and tuber crops, cocoyam is the considered as the third in importance in Nigeria (Olayiwola et al. 2013). Cocoyam is a rich carbohydrate source for human and animals (Nyochembeng and Garton 1998) and is an important cash crop for farmers (Tambong et al. 1997). Cocoyam corm is usually consumed after boiling, frying, or roasting and the corms can also be dried to make flour. In spite of its importance as a staple food in many countries, cocoyam is still an insufficiently studied and underutilized (Watanabe 2002). Efforts also need to be made and measures needs to be put in place to popularize, enhance and increase its use as food among the populace (Adejumo and Bamidele 2012) through the adaptation of technologies such as fermentation and parboiling that has been used to increase the utilization of other tubers such as yam and cassava.

*Lafun* is a the name of the fermented flour product obtained after peeling cassava, fermenting for 3–4 days, dewatering, drying, and milling (Adebayo‐Oyetoro et al. 2012). Yams slices are also parboiled in hot water (50°C for 3 h), steeped for 24 h, dried, milled to flour to obtain *elubo* (Babajide et al. 2006a,b). Both *lafun* are *elubo* are important delicacies in Nigeria where they usually made into paste and consumed with soup. With cocoyam being a tuber and root crop together with cassava and yam, which is used to produce *lafun* and *elubo*, respectively, there is need to also subject cocoyam to the same processing methods used in preparation of *lafun* and *elubo* (fermentation and parboiling) in order to evaluate the quality of these products. This can in turn increase the utilization of cocoyam being an underutilized crop.

Research has also shown that various biochemical and physiological changes are known to occur during storage of food, which can either influence or affect food quality (Tschannen 2003). Therefore, the evaluation of the quality changes during the storage of food products is of uttermost importance to establish the best storage condition for the product. Also, it is a well‐known that the storage stability of food systems depends on the storage conditions and information on the influence of storage conditions on quality of cocoyam flour is scarce in literature. The aim of this research was to assess the quality of flours produced from cocoyam and to evaluate the impact of different storage conditions on the quality of the flours. To achieve this cocoyam tuber were processed in ways similar to *lafun* as fermented cocoyam flour and *elub*o as parboiled cocoyam flour and the nutritional, functional, pasting properties, and pH of the flours were determined.

## Materials and Methods

### Materials

Cocoyam tubers used for this study were purchased from a local market in Abeokuta, Nigeria.

#### Production of cocoyam flours

##### Fermented cocoyam flour

Cocoyam flour was prepared according to the method of (Adebayo‐Oyetoro et al.*,* 2012) for the production of cassava flour (*Lafun*). Fresh cocoyam roots were peeled and then steeped for 3–4 days and allowed to ferment, the fermented roots were broken up into small pieces and dried at 50°C for 8 h before milling into flour.

##### Parboiled cocoyam flour

Cocoyam flour was prepared according to the method of (Babajide et al. 2006a,b) for the production of cassava flour (*elubo*). Fresh cocoyam roots were peeled, parboiled in hot water (50°C for 3 h) and then steeped for about 24 h and dried at 50°C for 8 h before milling into flour.

##### Storage Procedure

The flours samples were packaged in a high‐density polyethylene (HDPE) bags and stored at a relative humidity of 76% over three temperatures (25 ± 2, 35 ± 2, and 45 ± 2)°C for 8 weeks. Sample stored at room temperature (25 ± 2°C) in a cool dry place was used as control.

### Methods

The following analyses were carried out on the processed fermented and parboiled cocoyam flours samples for 8 weeks at 2 weeks interval starting from week 0.

#### Determination of Nutritonal composition

The cocoyam flour samples were analyzed for their proximate composition namely moisture, ash, fat, protein, and fiber using method number 950.46, 920.153, 991.36, 928.08, 993.21, respectively (AOAC, 2010). Carbohydrate was obtained by difference.

#### Determination of functional properties

##### Bulk density

This was evaluated by the method of Wang and Kinsella (1976).

##### Dispersibility

This was evaluated by the method described by Kulkarni and Ingle (1991).

##### Water absorption index

This was carried out using the modified method of Ruales et al. (1993).

##### Pasting properties

Pasting properties was determined using Rapid Visco Analyzer (RVA) (Model RVA 3D+; Network Scientific, Australia). Values for setback, breakdown, peak time, trough, pasting temperature, peak, and final viscosity were obtained from the pasting profile through the use of the Thermocline for Windows Software connected to a computer.

##### *Determination of physiochemical properties*

pH: The pH values of the flour were carried out using AACC, (2000) method.

### Statistical analysis

All procedures were carried out in triplicates and data collected from the study were subjected to multiple analysis of variance (MANOVA), significances were accepted at 5% level (*P* ≥ 0.05). The statistical software used was SPSS version 16.0 for windows (SPSS Inc., Chicago, IL, USA).

## Results

Table 1 shows the proximate composition of cocoyam flour as influenced by storage periods, processing methods, and storage temperatures. The moisture content, protein, fat, ash, crude fiber, and carbohydrate ranged from 9.34–13.5%, 3.55–7.04%, 2.23–3.79%, 3.06–6.99%, 2.50–4.74%, and 66.68–74.51%, respectively. Multiple analysis of variance (MANOVA) of the proximate composition revealed that storage period and its interactive effect with the processing methods had significant (*P* < 0.05) effects on all the parameters except for the fat content of the cocoyam flour. Also, the interactive effect of storage period and storage temperature as well as that of the processing methods and storage temperature reduction and drying methods had a significant effect on the proximate composition except for the ash content. Also the interactive effects of the three factors were insignificant on the ash and fat content of the flour.

**Table 1:** Proximate composition of cocoyam flour as influenced by storage periods, processing methods, and storage temperatures

Storage period	Processing methods	Storage temperature(°C)	Moisture (%)	Ash (%)	Protein (%)	Crude fiber (%)	Fat (%)	Carbohydrate (%)
Week 0	Fermentation	25	9.50 ± 0.34	3.33 ± 0.01	6.20 ± 0.04	3.50 ± 0.00	3.34 ± 0.04	74.13 ± 2.34
		35	9.45 ± 0.45	3.23 ± 0.04	6.00 ± 0.34	3.59 ± 0.23	3.56 ± 0.34	74.17 ± 0.45
		45	9.43 ± 0.49	3.06 ± 0.45	6.17 ± 0.10	3.32 ± 0.34	3.79 ± 0.12	74.23 ± 2.59
	Parboiling	25	10.50 ± 0.55	3.96 ± 0.00	6.97 ± 0.34	4.90 ± 0.23	3.43 ± 0.02	70.24 ± 0.34
		35	10.10 ± 1.34	3.98 ± 0.23	6.85 ± 0.06	4.74 ± 0.12	3.45 ± 0.12	70.88 ± 0.35
		45	9.34 ± 0.43	3.98 ± 0.10	6.74 ± 0.09	4.65 ± 0.45	3.45 ± 0.31	71.84 ± 1.34
Week 2	Fermentation	25	13.50 ± 0.32	6.67 ± 0.03	7.04 ± 0.04	3.00 ± 0.02	3.11 ± 0.02	66.68 ± 2.54
		35	12.00 ± 1.00	3.33 ± 0.10	6.25 ± 0.04	3.50 ± 0.20	3.34 ± 0.20	68.79 ± 2.32
		45	11.50 ± 0.00	6.66 ± 0.11	5.18 ± 0.03	4.50 ± 0.00	3.37 ± 0.11	68.79 ± 2.45
	Parboiling	25	13.00 ± 0.00	3.35 ± 0.23	4.18 ± 0.05	2.50 ± 0.23	2.46 ± 0.03	74.51 ± 2.43
		35	12.50 ± 0.50	3.33 ± 0.00	5.30 ± 0.01	4.50 ± 0.83	2.55 ± 0.11	71.82 ± 0.33
		45	12.00 ± 0.12	3.33 ± 0.05	6.25 ± 0.00	3.50 ± 1.00	2.47 ± 0.00	72.45 ± 0.24
Week 4	Fermentation	25	13.00 ± 0.05	6.66 ± 0.01	5.16 ± 0.05	2.50 ± 0.01	3.35 ± 0.01	69.33 ± 2.34
		35	11.53 ± 0.57	6.67 ± 0.41	4.18 ± 0.23	4.00 ± 0.17	3.36 ± 0.10	70.26 ± 1.31
		45	11.23 ± 0.05	6.66 ± 0.00	6.08 ± 0.34	2.50 ± 0.23	3.27 ± 0.21	70.26 ± 0.03
	Parboiling	25	12.50 ± 0.10	3.34 ± 0.01	5.36 ± 0.01	4.00 ± 0.00	2.73 ± 0.00	72.02 ± 1.44
		35	11.93 ± 0.12	3.36 ± 0.01	4.44 ± 0.11	4.50 ± 0.00	2.56 ± 0.00	73.21 ± 0.00
		45	11.50 ± 0.00	6.65 ± 0.20	4.49 ± 0.14	5.00 ± 0.00	2.23 ± 0.00	70.13 ± 0.28
Week 6	Fermentation	25	12.02 ± 0.21	6.97 ± 0.06	3.69 ± 0.87	3.50 ± 0.45	3.16 ± 0.28	70.66 ± 3.58
		35	11.39 ± 0.23	6.95 ± 0.12	3.33 ± 0.01	2.50 ± 0.03	3.32 ± 0.72	72.51 ± 2.89
		45	10.45 ± 0.23	6.99 ± 0.42	4.36 ± 0.44	3.00 ± 0.53	3.52 ± 0.75	71.68 ± 1.99
	Parboiling	25	10.80 ± 1.00	6.67 ± 0.01	7.04 ± 0.05	4.50 ± 0.55	2.56 ± 0.09	68.43 ± 0.23
		35	10.40 ± 0.50	6.66 ± 0.01	5.40 ± 0.01	3.50 ± 0.00	2.52 ± 0.87	71.52 ± 0.02
		45	10.09 ± 0.00	6.68 ± 0.10	4.97 ± 0.34	3.00 ± 0.34	2.62 ± 0.44	72.64 ± 2.36
Week 8	Fermentation	25	11.74 ± 0.46	6.96 ± 1.02	4.80 ± 0.69	4.50 ± 0.45	3.23 ± 0.03	68.77 ± 0.24
		35	10.98 ± 0.02	6.99 ± 0.10	5.40 ± 0.02	3.50 ± 0.50	3.27 ± 0.03	69.86 ± 1.71
		45	9.50 ± 0.50	6.98 ± 0.02	6.46 ± 0.12	4.00 ± 1.00	3.25 ± 0.72	73.86 ± 1.82
	Parboiling	25	10.00 ± 0.34	6.98 ± 0.30	3.55 ± 0.03	3.50 ± 0.29	2.61 ± 0.32	73.36 ± 0.98
		35	10.29 ± 0.62	6.93 ± 0.01	6.46 ± 0.13	4.50 ± 0.23	2.37 ± 0.33	69.45 ± 1.39
		45	9.34 ± 0.34	6.99 ± 0.29	4.57 ± 0.12	3.00 ± 0.50	2.52 ± 0.41	71.58 ± 2.23
Range			9.34 ± 0.34–13.5 ± 0.32	3.06 ± 0.45–6.99 ± 0.29	3.55 ± 0.03–7.04 ± 0.05	2.50 ± 0.23–4.74 ± 0.12	2.23 ± 0.00–3.79 ± 0.12	66.68 ± 2.54–74.51 ± 2.43
Mean			11.05	5.31	5.43	3.72	2.99	71.23
SD			0.36	0.15	0.16	0.50	0.22	1.71
SE			0.07	0.03	0.03	0.09	0.04	0.31
p of storage period (S)			***	***	***	***	ns	***
p of processing method (P)			***	***	ns	***	***	ns
p of storage temperature (T)			***	ns	***	***	ns	***
p of S × P			***	***	***	***	ns	***
p of S × T			***	ns	***	***	***	***
p of P × T			***	ns	***	***	***	***
p of S × P × T			***	ns	***	***	ns	***

Results are expressed as mean ± standard deviation. ns denotes parameters that are not significantly different at 5% confidence level (*P* > 0.05), whereas *** denotes values that are significantly different at 5% confidence level (*P* < 0.05). SD denotes standard deviation, SE denotes standard error of mean, whereas *P* denotes probability.

The influence of processing methods, storage period, and temperature on the functional properties of cocoyam flour is shown in Table 2. Water absorption index, wettability, dispersibility, and bulk density ranged from 1.94–2.72%, 25.30–135.50 sec, 80.29–86.50%, and 0.48–0.71 g/cm^3^, respectively. The wettability of the fermented cocoyam flour samples increased as the storage temperature at each storage interval and the parboiled cocoyam floured stored for 4 weeks at 45°C had the highest value for wettability. Processing methods and storage period had significant effect on the wettability and bulk density of the samples, whereas storage temperature and the interactive effect of the processing methods and storage period had no significant effect on dispersibility and bulk density of the samples. Also processing methods, storage period and temperature had no significant effect on the dispersibility, bulk density, and water absorption index of the samples, but its effect was significant for wettability (Table 2).

**Table 2:** The functional properties of cocoyam flour as influenced by storage periods, processing methods, and storage temperatures

Storage period	Processing methods	Storage temperature(°C)	Wettability (sec)	Dispersibility (%)	Bulk density (g/cm^3^)	Water absorption Index	Water binding capacity
Week 0	Fermentation	25	81.50^ ^± 0.50	80.29^ ^± 0.50	0.70^ ^± 0.01	1.98^ ^± 0.02	198.40 ± 6.00
		35	82.50^ ^± 1.03	81.23^ ^± 2.36	0.70^ ^± 0.00	1.98^ ^± 0.20	198.70 ± 0.61
		45	81.50^ ^± 1.50	81.50 ± 1.00	0.70^ ^± 0.01	1.98^ ^± 0.10	198.32 ± 10.00
	Parboiling	25	26.50 ± 1.50	83.50^ ^± 5.00	0.63^ ^± 0.01	2.40^ ^± 0.00	240.15 ± 5.22
		35	26.60 ± 0.05	84.30^ ^± 1.04	0.63^ ^± 0.03	2.40^ ^± 0.12	240.15 ± 5.23
		45	25.30^ ^± 2.73	86.40^ ^± 0.22	0.63^ ^± 0.01	2.40^ ^± 0.21	240.15 ± 4.53
Week 2	Fermentation	25	63.50^ ^± 0.50	82.50^ ^± 2.43	0.59^ ^± 0.02	1.98^ ^± 0.10	198.45 ± 7.43
		35	63.50^ ^± 1.04	81.50^ ^± 2.00	0.61^ ^± 0.01	1.96^ ^± 0.02	196.65 ± 4.33
		45	97.50^ ^± 2.50	82.00^ ^± 0.34	0.61^ ^± 0.04	2.10^ ^± 0.21	210.45 ± 3.45
	Parboiling	25	122.50^ ^± 0.50	85.50^ ^± 3.00	0.64^ ^± 0.12	1.94^ ^± 0.03	194.50 ± 5.00
		35	84.50^ ^± 3.04	81.50^ ^± 1.03	0.65^ ^± 0.04	1.97^ ^± 0.10	196.95 ± 2.54
		45	107.50^ ^± 2.73	83.50^ ^± 2.37	0.64^ ^± 0.03	2.10^ ^± 0.01	210.50 ± 10.25
Week 4	Fermentation	25	63.50^ ^± 3.01	83.00^ ^± 3.00	0.63^ ^± 0.05	2.03^ ^± 0.05	203.00 ± 6.50
		35	65.00^ ^± 5.03	84.00^ ^± 4.00	0.64^ ^± 0.12	2.11^ ^± 0.41	210.90 ± 1.16
		45	69.00^ ^± 1.00	84.50^ ^± 0.76	0.64^ ^± 0.34	2.13^ ^± 0.21	213.00 ± 8.17
	Parboiling	25	135.00^ ^± 1.00	83.00^ ^± 3.00	0.63^ ^± 0.51	2.00^ ^± 0.28	200.20 ± 0.15
		35	96.00^ ^± 0.33	84.00^ ^± 0.66	0.64^ ^± 0.00	1.97^ ^± 0.03	197.25 ± 0.62
		45	135.50 ± 0.05	86.00^ ^± 1.73	0.67^ ^± 0.00	1.98^ ^± 0.46	198.30 ± 5.67
Week 6	Fermentation	25	56.00^ ^± 2.08	82.50^ ^± 0.57	0.53^ ^± 0.00	2.18^ ^± 0.04	218.30 ± 1.73
		35	67.50^ ^± 6.02	84.50^ ^± 0.38	0.50^ ^± 0.00	2.33^ ^± 0.00	232.95 ± 11.91
		45	86.00^ ^± 2.81	85.50^ ^± 0.58	0.53 ± 0.08	2.43^ ^± 0.05	242.80 ± 5.85
	Parboiling	25	133.00^ ^± 1.53	86.00^ ^± 3.00	0.59^ ^± 0.10	2.07^ ^± 0.07	207.40 ± 4.80
		35	91.50^ ^± 0.29	83.50^ ^± 1.69	0.63^ ^± 0.03	1.97^ ^± 0.25	197.00 ± 0.58
		45	91.50^ ^± 1.00	86.50^ ^± 0.41	0.56^ ^± 0.03	2.03^ ^± 0.25	202.70 ± 1.69
Week 8	Fermentation	25	52.00^ ^± 3.35	82.00^ ^± 1.83	0.48^ ^± 0.02	2.54^ ^± 0.26	253.60 ± 2.66
		35	64.50^ ^±0.50	82.00^ ^± 0.58	0.54^ ^± 0.00	2.67^ ^± 0.01	266.90 ± 7.93
		45	38.00^ ^±1.54	83.50^ ^± 0.08	0.52^ ^± 0.00	2.72^ ^± 0.13	272.35 ± 2.34
	Parboiling	25	120.00^ ^± 0.76	85.50^ ^± 1.23	0.59^ ^± 0.01	2.05^ ^± 0.05	205.30 ± 2.86
		35	84.00^ ^± 1.33	86.40^ ^± 0.18	0.54 ± 0.23	2.05^ ^± 0.08	205.15 ± 3.30
		45	77.50^ ^± 2.45	85.40^ ^± 0.18	0.59^ ^± 0.00	2.07 ±0.23	206.75 ± 3.01
Range			25.30^ ^± 2.73–135.50 ± 0.05	80.29 ± 0.50–86.5 ± 0.41	0.48 ± 0.02–0.71 ± 0.00	1.94 ± 0.03–2.72 ± 0.13	194.50 ± 5.00–272.35 ± 2.34
Mean			79.60	83.91	0.63	2.15	215.74
SD			1.75	1.51	0.06	0.13	4.52
SE			0.32	0.28	0.01	0.03	0.83
p of storage period (S)			***	ns	***	***	ns
p of processing method (P)			***	ns	***	ns	***
p of storage temperature (T)			***	ns	ns	ns	ns
P of S × P			***	ns	ns	***	ns
p of S × T			***	***	ns	ns	ns
p of P × T			***	ns	ns	***	ns
p of S × P × T			***	ns	ns	ns	ns

Results are expressed as mean ± standard deviation. ns denotes parameters that are not significantly different at 5% confidence level (P > 0.05), whereas *** denotes values that are significantly different at 5% confidence level (*P* < 0.05). SD denotes standard deviation, SE denotes standard error of mean, whereas *P* denotes probability.

Table 3 shows the results of the pasting properties of cocoyam flour samples which were subjected to different processing methods and stored under different temperatures for different duration. The peak viscosity, trough, breakdown, final, and setback viscosity of the samples ranged from 178.05–380.40, 131.90–298.00, 26.80–89.50, 204.60–424.75, 100.00–935.50, RVU, respectively. The peak time and pasting temperature ranged from 5.04–5.27 min and 69.18–85.40°C, respectively. The temperature of storage significantly affected all the pasting properties except the peak time and breakdown viscosity. All the pasting properties were significantly affected by the storage period, whereas the interaction between storage temperature, processing methods, and storage period affected all the pasting properties except the peak time, peak, and breakdown viscosity.

**Table 3:** The pasting properties of cocoyam flour as influenced by storage periods, processing methods, and storage temperatures

Storage period	Processing methods	Storage temperature(°C)	Peak viscosity	Through	Breakdown	Final viscosity	Setback	Peak time	Pasting temperature
Week 0	Fermentation	25	245.20 ± 0.68	228.70 ± 2.48	31.35 ± 1.03	284.55 ± 2.13	101.20 ± 3.21	5.21 ± 0.15	83.55 ± 2.41
		35	231.20 ± 5.05	212.70 ± 2.34	31.35 ± 1.23	280.950 ± 1.23	104.00 ± 10.32	5.71 ± 0.12	82.67 ± 0.35
		45	223.40 ± 1.03	228.70 ± 2.13	31.35 ± 0.54	270.50 ± 1.35	101.10 ± 2.23	5.17 ± 0.17	83.98 ± 0.34
	Parboiling	25	349.55 ± 3.24	253.25 ± 3.42	81.70 ± 0.77	317.20 ± 1.36	121.53 ± 8.86	5.06 ± 0.45	84.30 ± 0.23
		45	332.55 ± 2.34	253.25 ± 2.16	81.70 ± 0.45	309.30 ± 1.26	123.91 ± 1.10	5.14 ± 0.54	83.90 ± 0.12
		45	314.75 ± 3.45	253.25 ± 1.86	81.70 ± 0.35	297.30 ± 0.35	120.90 ± 5.19	5.18 ± 0.11	84.44 ± 2.10
Week 2	Fermentation	25	2124.50 ± 0.62	181.65 ± 2.48	30.80 ± 1.45	271.00 ± 0.36	893.50 ± 20.32	5.20 ± 0.23	84.45 ± 1.00
		35	198.05 ± 0.60	131.90 ± 0.57	30.65 ± 0.68	255.95 ± 1.95	922.00 ± 23.12	5.17 ± 0.11	73.53 ± 1.43
		45	178.05 ± 4.53	150.25 ± 0.46	27.80 ± 0.33	204.60 ± 0.97	543.50 ± 12.34	5.27 ± 0.02	84.78 ± 0.05
	Parboiling	25	360.75 ± 1.72	274.00 ± 1.73	86.95 ± 1.96	354.95 ± 1.55	115.35 ± 2.45	5.10 ± 0.42	84.40 ± 1.07
		35	368.35 ± 2.45	199.90 ± 1.48	79.25 ± 0.34	414.45 ± 1.37	125.35 ± 4.56	5.20 ± 0.45	84.75 ± 0.05
		45	373.45 ± 5.63	276.65 ± 0.78	96.80 ± 0.42	412.30 ± 1.43	135.65 ± 2.98	5.07 ± 0.45	84.35 ± 0.74
Week 4	Fermentation	25	215.50 ± 5.03	184.25 ± 0.87	31.25 ± 1.32	263.65 ± 2.99	794.00 ± 10.34	5.24 ± 0.56	69.18 ± 0.75
		35	232.90 ± 3.06	195.20 ± 2.57	37.70 ± 0.33	288.75 ± 0.96	935.50 ± 8.00	5.14 ± 0.13	84.33 ± 0.53
		45	212.60 ± 1.72	176.05 ± 1.00	36.55 ± 0.70	237.80 ± 1.35	617.50 ± 4.97	5.17 ± 0.34	85.60 ± 0.80
	Parboiling	25	350.10 ± 0.65	260.60 ± 0.61	89.50 ± 0.68	396.60 ± 1.15	126.60 ± 2.53	5.07 ± 0.53	83.98 ± 2.31
		35	366.30 ± 1.18	293.10 ± 1.01	73.20 ± 1.23	407.80 ± 1.56	114.70 ± 3.07	5.23 ± 0.28	85.20 ± 2.08
		45	358.55 ± 1.71	277.25 ± 0.78	81.30 ± 1.36	419.90 ± 1.85	142.65^ij^± 4.82	5.07 ± 0.34	84.00 ± 2.19
Week 6	Fermentation	25	201.10 ± 1.88	174.30 ± 0.65	26.80 ± 1.02	265.40 ± 1.43	911.50 ± 23.53	5.13 ± 0.00	83.13 ± 1.67
		35	233.55 ± 1.73	203.10 ± 0.39	30.45 ± 0.24	309.80 ± 1.16	106.70 ± 5.54	5.13 ± 0.34	84.00 ± 1.74
		45	201.40 ± 1.92	163.00 ± 0.27	38.40 ± 0.20	226.40 ± 1.10	634.00 ± 9.67	5.13 ± 0.17	85.20 ± 1.88
	Parboiling	25	380.40 ± 2.14	293.55 ± 0.65	86.85 ± 1.34	423.85 ± 1.15	130.30 ± 5.34	5.04 ± 0.45	83.98 ± 1.51
		35	368.75 ± 0.42	298.00 ± 0.38	70.75 ± 1.97	424.75 ± 3.35	126.60 ± 4.34	5.20 ± 0.45	84.88 ± 0.98
		45	225.20 ± 2.56	228.70 ± 0.38	35.35 ± 1.24	271.15 ± 0.85	100.00 ± 2.45	5.21 ± 0.34	84.55 ± 0.45
Week 8	Fermentation	25	225.20 ± 1.06	228.70 ± 3.95	32.35 ± 0.89	271.15 ± 1.93	100.00 ± 5.63	5.17 ± 0.14	83.65 ± 2.99
		35	225.200 ± 3.44	228.70 ± 1.24	30.35 ± 0.65	271.15 ± 0.45	100.11 ± 4.56	5.27 ± 0.56	83.55 ± 0.32
		45	342.55 ± 2.19	253.25 ± 1.83	81.70 ± 1.73	307.30 ± 1.84	119.00 ± 3.56	5.10 ± 0.65	85.40 ± 0.12
	Parboiling	25	342.55 ± 0.15	258.35 ± 1.49	82.7.00 ± 0.43	307.30 ± 2.35	121.90 ± 4.56	5.20 ± 0.02	84.00 ± 0.15
		35	342.55 ± 2.67	234.35 ± 1.57	80.7.00 ± 1.67	307.30 ± 0.21	120.90 ± 3.54	5.10 ± 0.23	84.40 ± 0.34
		45	212.45 ± 3.43	181.65 ± 1.26	30.8.00 ± 0.34	271.00 ± 1.26	893.50 ± 8.32	5.20 ± 0.04	84.45 ± 0.23
Range			178.05 ± 4.53–380.40 ± 2.14	131.90 ± 0.57–298.00 ± 0.33	26.80 ± 1.02–89.50 ± 0.68	204.6.00 ± 0.97–424.7.5 ± 3.35	100.00 ± 2.45–9.35.50 ± 8.00	5.04 ± 0.45–5.27 ± 0.56	69.18 ± 0.75–85.60 ± 0.12
Mean			280.80	225.88	55.61	311.44	320.12	5.18	83.42
SD			2.29	1.41	0.89	1.52	7.04	0.29	1.76
SE			0.42	0.26	0.16	0.28	1.29	0.05	0.32
p of storage period (S)			***	***	***	***	***	***	***
p of processing method (P)			ns	***	***	***	***	***	***
p of storage temperature (T)			***	***	ns	***	***	ns	***
p of S × P			***	***	***	***	***	ns	***
p of S × T			ns	***	***	ns	***	***	***
p of P × T			ns	***	ns	ns	***	ns	***
p of S × P × T			ns	***	ns	***	***	ns	***

Results are expressed as mean ± standard deviation. ns denotes parameters that are not signifcantly different at 5% confidence level (P > 0.05), whereas ***denotes values that are significantly different at 5% confidence level (P < 0.05). SD denotes standard deviation, SE denotes standard error of mean, whereas P denotes probability.

**Figure 1:**
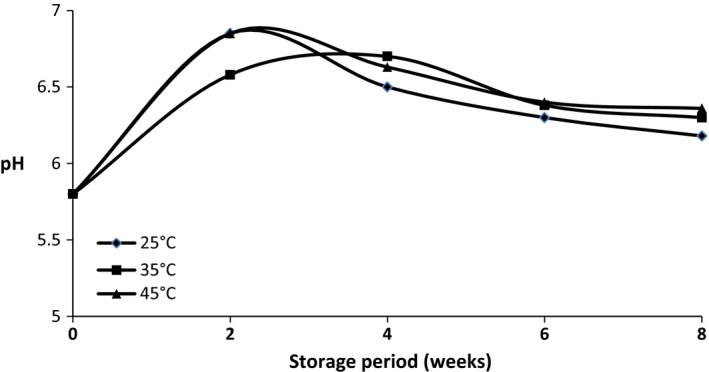
Changes in the pH of parboiled cocoyam stored at 25°C, 35°C, 45°C for 8 weeks at 2 weeks interval.

**Figure 2:**
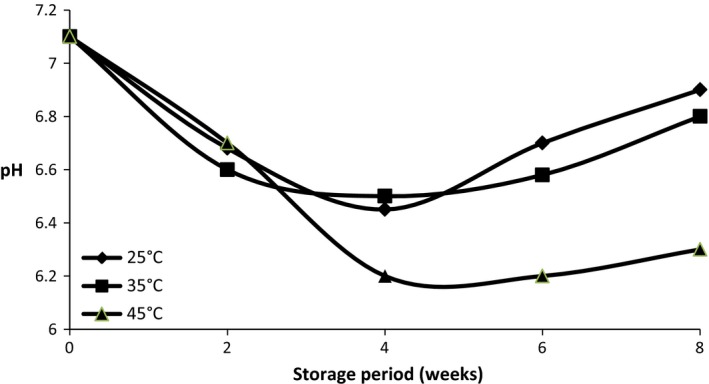
Changes in the pH of fermented cocoyam stored at 25°C, 35°C, 45°C for 8 weeks at 2 weeks interval.

Figure 1 and 2 shows the pH of cocoyam flour samples (fermented and parboiled, respectively) stored at 25°C, 35°C and 45°C for 8 weeks. The pH of the fermented cocoyam flour ranged from 5.8 to 6.92, whereas the pH of the unfermented but parboiled cocoyam flour ranged from 6.23 to 7.12. The pH of the samples fermented cocoyam flour increased at the beginning of the storage period and later reduced while that of the unfermented but parboiled sample reduced and later increased within the storage period.

## Discussion

It is a well‐known that the storage stability of food systems depends on storage conditions such as length and time of storage and information on the influence of storage conditions on quality of food products is of uttermost importance. This study investigated the quality changes that occur during the storage of cocoyam flour samples. Moisture content is an indicator of shelf stability; increase in moisture content enhances microbial contamination and reduces food quality and stability (Akanbi et al. 2009), therefore the lower the moisture content of a sample, the more its storability and proficiency for industrial processing. Values obtained for moisture content in this study are higher than the ones reported for of the cocoyam flour samples in a study by Ogunlakin et al. (2012) who evaluated the proximate composition and physiochemical properties of yam during drying. Also, storage temperature and storage period and their interactive effect were significant on the moisture content of the samples. The cocoyam flour samples were stored at different temperatures and for different periods and this might be responsible for the observed significance. The recommended safe level of moisture content during storage of flours or food powder is between 12 to 14% (FAO, 1993) and the values obtained in this study fell between this range.

Proteins and carbohydrates are some of the basic constituents of the consumable part of plant that determines its nutritional value. The protein content of all samples was relatively low and cocoyam has been reported as a poor source of protein (Oyenuga 1992; Okaka and Isieh 2002). Processing methods (fermentation and parboiling) had significant effect on the protein content of the flour. Processing techniques such as fermentation has been reported to increase protein content of products (Nnam 2000; Enujiugha 2003), whereas parboiling has been found to decrease protein content due to leaching of nitrogenous substances during soaking and rupturing of molecules during steaming (Otegbayo et al. 2001) and this might be the reason for the observed effect. The high content of carbohydrate observed in the samples may be attributed to the high amount of carbohydrate in cocoyam.Enwere (1998) reported that, carbohydrate is the major nutrients in tuber and root crops. Carbohydrates assist in the metabolism of fat and are known to supply of quick and metabolizable energy in the body. In this study, drying methods had significant effect on the carbohydrate content of the flour. The results of the carbohydrate content were within the range of those reported by Igbabul et al. (2014) for cocoyam flour.

Fat is of uttermost importance in diets as it promotes the absorption of fat‐soluble vitamins and plays a significant role in the shelf life of food products (Bogert et al. 1994). High fat content in foods can promote rancidity that leads to the development of off flavor (Ihekoronye and Ngoddy 1985). As such, relatively low‐fat content, as values obtained for cocoyam in this study are desirable in baked food products. The flour from the parboiled, 45°C and week 4 stored sample had the lowest fat content and this implies that will be able to withstand a longer storage period than other samples. The significant effect observed because of the processing methods could be due to the activities of lipolytic enzymes and that of the fermenting microflora during storage. A reflection of the minerals contained in a food sample is its ash content. Baah (2009) reported that the amount of ash in a tuber depends on the type of soil from which it was harvested, the moisture content and maturity of the crop. The significant effect of processing method, storage period, and storage temperature on cocoyam flour could be due to leaching of soluble mineral elements and enzymatic activities of the fermenting microorganisms. Atti (2000) on reported a decrease in ash content in fermented millet, whereas SEFA–DEDEH and Kluvitse (1995)observed an increase in ash content in fermented maize cowpea blends, both increase and decrease were observed in the ash content of the cocoyam flour studied.

The quantity of sugars that cannot be digested which are present in a food sample is its crude fiber; crude fiber has been found to support the movement of food across the digestive tract (Adeleke and Odedeji 2010). High crude fiber aids the bulk density of foods, which could help in the movement of bowel, lowering of blood cholesterol, and prevention of colon cancer. The flour of the 35°C parboiled sample had more than 3% fiber and this implies that it will serve as a good source of fiber than the other samples. All the factors had significant effect on the cocoyam flour samples. Crude fiber content decreased from 0.73 to 0.19% and this can be due to softening of the fibrous tissues and the bio‐conversion of carbohydrates and lignocelluloses into protein Hwei‐Ming et al. (1994), formation of adducts from non‐structural carbohydrates as well as polymerization and fragmentation of cell wall monosaccharides (Nelson and Bozich 1996).

The functional properties of the food materials are very important for the suitability of diet particularly for the growing children (Omueti et al. 2009). The water absorption index quantifies the portion which starch occupies after it swells in surplus water (Mason and Hoseney 1986; Adeleke and Odedeji 2010) and this can a gelatinization index (Atlan et al.*,* 2008). Processing methods had no significant effect on the water absorption index of the samples. Several studies have reported the enhancement of water absorption index of different cereals through different processing conditions, for example, parboiling (Mariotti et al. 2005). Higher values of wettability indicate lower reconstitution properties therefore the parboiled sample stored at 45°C for 4 weeks will be the slowest to reconstitute than the other samples. Storage temperature had significant effect on the wettability of the cocoyam flour samples. During a process that requires the application of heat some of the starch present in flour may have gelatinized and in process absorb moisture, swell and consequently lead to reduced wettability and this might be the reason for the observed effect.

Shittu et al. (2005) reported bulk density as an important parameter that determines the transportation and packaging demands of food products. Bulk density of the cocoyam flours were reduced during storage and the highest value was before storage (0.70 g/cm^3^). Oladeji et al. (2013) reported the value of bulk density of cocoyam flour to be 0.71 g/cm^3^. It is desirable to have bulk density that gives higher packaging benefit, as more flour will be packed within a constant space (Adepeju et al. 2011). Therefore, in this study the week 0 fermented and parboiled samples will offer a greater packaging advantage than other samples since it has the highest bulk density value. Highest value of dispersibility was observed in the parboiled cocoyam flour sample that was stored at 45°C for 6 weeks hence, will make them to be easily reconstituted to fine dough during mixing than other samples.

Pasting characteristics of flour are of importance in food processing because they influence the texture, stability, and digestibility of starchy foods and, thus, determine the application and use of the flour in various food products. The peak viscosity values obtained in this study is relatively high compared to the range of values reported for cocoyam flour 161.80–267.58 RVU by Ejoh et al. (2013). The lowest value was obtained in the fermented sample stored at 45°C for 2 weeks and the highest in the parboiled sample stored at 25°C for 6 weeks. This parameter reveals the starch's ability to freely swell before breaking down physically (Sanni et al. 2004). The significant effect of temperature observed on the peak viscosity of the cocoyam flour samples investigated, may influence their performance during product development. Trough quantifies the ability of starch to stay undamaged when flour is subjected to a constant and high temperature for a long period of time during steaming. The sample parboiled and stored at 35°C for 6 weeks had the highest trough and it is an indication that it has a greater tendency to resist shearing at elevated temperatures and high cooking paste stability (Farhat et al. 1999).

Peak time estimates the cooking time of the flour samples, the combined effects of processing methods, storage temperatures and period size had no significant effect on the flour samples as they were within a very close range. Oke and Bolarinwa, (2012) also reported values between 62.84 and 66.96RVU for cocoyam flour samples in their work on the effect of fermentation cocoyam flour properties. In this study, breakdown viscosities for the fermented cocoyam flour samples are between 26.80 and 38.40 RVU. Final viscosity is the change in the viscosity after holding cooked starch at 50°C and it represents cooked starch stability. Osungbaro et al. (2010) reported that final viscosity is the most frequently used factor to ascertain the quality of starchy food. The value, 167.46 RVU, obtained for final viscosity of cocoyam flour (unfermented) by Oke and Bolarinwa (2012), is lower than the values obtained for cocoyam samples in this study.

The temperature of pasting is an indication of the cooking temperature of the flour and indicates the probability of scorching. In most cases, high pasting temperature leads to scorching before a paste is well cooked. Hence, there is need to continuously stir the food sample that have high pasting temperatures during cooking. Pasting temperature of the cocoyam flour samples investigated was higher than those reported by Oladeji et al. (2013). In the study of Ejoh et al. (2013), pasting temperatures ranged from 61.73 ± 0.04 to 61.95 ± 0.42°C and are lower than the values obtained in this study. Since fermentation and storage at 45°C for 4 weeks resulted into sample with the highest pasting temperature, it suggests that the resultant flour from this process will cook at a higher temperature than other samples and hence it require higher energy and will cook for a longer period than the other samples.

The acidity or alkalinity level of a substance is its pH, in this study, the pH of the fermented cocoyam flour increased at week 2 and gradually decreased until the end of the storage period (week 8). In a study by Rehman (2004), a significant decrease in pH of wheat, rice and maize grains was observed at 25 and 45°C storage temperatures. Also, the pH of the parboiled cocoyam flour reduced and later increased. The varied pH values observed in the samples during storage may be due to the activities of microorganisms during the processing of the cocoyam flours as observed by Enujiugha (2003), Teniola (1990).

## Conclusion

This study showed that processing methods (fermentation and parboiling), storage period (0, 2, 4, 6, 8 weeks), and storage temperatures (25°C, 35°C, 45°C) produced different characteristics of cocoyam flour. All the parameters had effect on moisture, crude fiber, wettability, trough, setback and pasting temperature of the flour. Their interactive effects were significant on protein, carbohydrate but insignificant on bulk density and the water binding capacity of the flour. Disparities were also observed in the pH levels of the cocoyam flour samples.

## Conflict of Interest

None declared.
